# Supplementary material for: Using natural vegetation succession to evaluate how natural restoration proceeds under different climate in Yunnan, Southwest China
Source: PLoS One. 2025 Mar 11;20(3):e0319420. doi: 10.1371/journal.pone.0319420 (PMC11896063; doi:10.1371/journal.pone.0319420)
Supplement: S3 Appendix — (DOC) [file pone.0319420.s003.doc]

1.The code for Fig.3, which to divide the region into sub-areas:

getwd()

df<-read.csv("test/twin.csv",header=T,row.names = 1)

library(ggtree)

head(df)

hc<-hclust(dist(df))

ggtree(hc,layout="circular"，size=0.5)+

geom_tiplab(offset=0.003,size=3,hjust=-8)+

theme_tree2()+

xlim(0,0.22) +

geom_text(aes(label=node))+ geom_highlight(node=63,fill="red",alpha=0.3)+

geom_highlight(node=74,fill="steelblue")+

geom_highlight(node=75,fill="green")+

geom_cladelabel(node=63,label="vc",offset=0.045,barsize=2,color="red",vjust=3,hjust =1)+ geom_cladelabel(node=74,label="VD",offset=0.045,barsize=2,color="steelblue",vjust=-0.5,hjust =4)+

geom_cladelabel(node=75,label="VE",offset=0.045,barsize=2,color="green",vjust=-0.5,hjust =-1)

str(hc)

2.The code for Fig.4, which to create the spatial variations of each habitat factors in each climate sub-areas

library(ggridges)

library(ggplot2)

library(viridis)

library(hrbrthemes)

ggplot(df, aes(x = `accumulate_temperature`, y = `zone`, fill = ..x..)) +

geom_density_ridges_gradient(scale = 2, rel_min_height = 0.13) +

scale_fill_viridis(name = "Temp. [F]", option = "C") +

labs(title = 'Accumulate temperatures in first division') +

theme_ipsum()+

theme(

legend.position="none",

panel.spacing = unit(0.1, "lines"),

strip.text.x = element_text(size = 8)

)

3.The code for Fig.5, which to create the time of each succession stage in each climate sub-areas

df<-read.csv("test/midone.csv",header=T)

head(df)

library(ggplot2)

ggplot(df, aes(x=group, y=year,fill=type))+

scale_y_continuous(limits=c(0, 35))+

geom_violin(trim=FALSE,color="white",width=1.2,position = position_dodge(0.8))+

stat_boxplot(geom="errorbar",width=0.15,position = position_dodge(0.8))+

geom_boxplot(width=0.1,position = position_dodge(0.8),outlier.colour = "transparent")+

scale_fill_manual(values = c("green", "orange","steelblue"))

4.The code for Fig.7, which to create the supplementary distribution in each climate sub-areas

library(ggplot2)

getwd()

df<-read.csv("test/compensate.csv",header = T)

head(df)

df$region<-factor(df$region,levels=c("H-I","H-IV","W-I","M-III","H-III","M-II","W-III","W-IV","C-II","W-II","M-I","H-II","C-I"))

ggplot(data=df,aes(x=region,y=resistance))+

geom_line(aes(group=group))+

geom_point()+

geom_hline(yintercept =2.0234,col = "green", lwd =1.2, lty = 2)+

theme(panel.background = element_blank(),axis.line = element_line())
