# Supplementary material for: Using natural vegetation succession to evaluate how natural restoration proceeds under different climate in Yunnan, Southwest China
Source: PLoS One. 2025 Mar 11;20(3):e0319420. doi: 10.1371/journal.pone.0319420 (PMC11896063; doi:10.1371/journal.pone.0319420)
Supplement: Attachment — Submitted filename: Revision Plos One.pdf [file pone.0319420.s004.pdf]

Using Spontaneous Vegetation Succession to Evaluate How Natural Restoration Works under Different Climate in Yunnan, Southwest China

The present work deals with the use of Spontaneous Vegetation Succession in China to Evaluate How Natural Restoration Works under Different Climate. First of all, if authors would like to present their study in a more scientific format, some title words should be replaced to more appropriate concepts. "Spontaneous vegetation" could be replaced to "herbaceous vegetation" or "successional vegetation" since the word "spontaneous" is not a functional or structural concept of vegetation. In a similar way, the word "works" is not a proper word to refer to ecosystem restoration activity. Perhaps the word "proceeds" or "progress" should adheres better.

The short title does not read short.

Keywords:

Natural restoration; Spontaneous vegetation succession: It is strongly recommended that there should not be the same title words but different ones contained in the document or refer to the study topic that helps to retrieve the paper in addition to the title words solely.

Climate condition sub areas; Index quantify; Community characteristic: these keywords are not clear or properly written or the authors have not clarity on the concepts they want to consider.

Abstract

"Yet, there remain uncertainties regarding with natural restoration, for example, how to confirm natural restoration works for different climate conditions, and how could we quantify it."

Natural restoration has been widely and for long time studied, formerly in temperate climates and there after in tropical and many other climates. Then, this motivation is not sufficiently strong.

However, the aim of quantifying Natural Succession from 1703 succession monitoring data(1987 to 2017) with habitat quality data (14 sub-areas) in different Seral stages(grassland, shrub and forest), is a novelty and interesting research.

The word "spontaneous" simply is herein used as a synonymy of the word "natural", so it could be better to use the longstanding term of "natural" used in this literature.

"The rule of it follows as H-I > H-IV > W-I > M-III > H-III > M-II > W-III > W-IV > C-II > WII > M-I > H-II > C-I. The times of spontaneous succession in forest stage from grassland stage in each sub-area are similar as the rule of index. The dominate species in each sub-area are different. The stand volumes are influenced by the index, and showed a positive correlation. Future, the index could be used to reallocate the investment in natural restoration project for better returns.

Constant vigilance is required in the first 5 years after restoration actions implemented to avoid failure from calculated error."

The above paragraph is not clear. It needs to be rewritten. The rule (letters and "numbers" or categories) shows there does not say where or how it comes from to many readers not specialized in this topic. After reading methodology and Results, I see a problem in summarizing the key results into the abstract. Authors should attend this observation to give the reader a comprehensible understanding of what these letters and categories mean.

The abstract does not show clearly the Results or the quantitative data obtained in the study. It only limits to three description lines without figure numbers. Perhaps provide ranges of data from Table 3. Stand volumes of vegetation are key results of regeneration and may be shown here.

Introduction

Line 41. At the end replace "M" for "m".

Line 45 – 53. Are not fluently reading.

Materials and methods

Line 89. The word "investigative" could be replaced for "research".

The number of shared characteristics of the samples are 4 or 5?

Line 116. It should be briefly explained how is annual accumulated temperature obtained though is not a common variable used. Is it a daily summarizing of temperature values (maximum day value or which temperature value) or how are the temperature values summarized throughout the year.

Could be useful to mention which graphical software was used to make figures 3-5.

Results

Line 202. Fig. 4. Should be better to delete "It's a figure to show the" and simply start the text with "Spatial variation of……".

Line 210, 211. Text already presented in Table 2, so decide which way to present Resistance and Habitat quality data, in a paragraph or a table. Rewrite the paragraph accordingly to the decision.

Table 3. Use of percent or percentage? Correct.

Table 4. Make shorter community characteristics column and expand the previous four columns for better reading.

Discussion

Line 292. Tang does not match with number [54]. Correct.

Use italics for the scientific names.
