# Supplementary material for: Using natural vegetation succession to evaluate how natural restoration proceeds under different climate in Yunnan, Southwest China
Source: PLoS One. 2025 Mar 11;20(3):e0319420. doi: 10.1371/journal.pone.0319420 (PMC11896063; doi:10.1371/journal.pone.0319420)
Supplement: Attachment — Submitted filename: response letter for reviewer2.docx [file pone.0319420.s005.docx]

Response to reviewer 2:

Dear professor，

Thanks for your precious advice. These advice possess great significance for improving the quality of our manuscript. We attached great importance to these, and took actions carefully to revise our manuscript. In this response letter we attached the advice for our manuscript and made the response under it. Furthermore, we resubmitted the revised manuscript to submission system, and the texts we had revised were marked in red in the new one. The details of modification below as:

The manuscript PONE-D-24-44569 contains important findings that contribute to our understanding of spontaneous or natural" vegetation succession across different climatic conditions in Yunnan, China. The findings will be of significant interest to the broad scientific community and are backed by a strong data set spanning 30 years of observations.However, this does not come through from the abstract nor the introduction, and it is hard to understand from the current presentation of the methods.

Q1:In order to better understand and appreciate the findings and implications of this study a better organization of the methods is needed, which then can be used to re-write the abstract and discussion. For example, the abstract does not do justice to the span of sites, observations, and intervals of the observation used in this study.Only after reading halfway into the manuscript is enough information presented on the 30-year data set that has 6 observations taken at five-year intervals.

A:Dear Professor, thank you for your advice. For abstract, we have rewrote this section, which include the motivation of why we conducted this research, more information to present the 30-year data set, the categories of sub-area where came from, and the detailed information of result(natural succession index value, range of succession time in each sub-areas, range of stand volume in each sub-areas, relations between them). For methods, we have revised this section, which include more information to show how we divided the study area into sub-areas and details of crude data in this research part, more details to show how to calculate the natural succession index, and more information of observation system (observations, details of quadrat, intervals of observation, span of sites). For discussion, we have rewrote this section, include some unreasonable paragraph structures, some inconsistent sentences, and some vague expressions.

Q2:The authors are suggested to please improve readability of the text narrative by providing information on the study design and how locations-sites/plots/samples/time intervals. As it is currently presented it does not convey the importance and complexity of the results being present, and it even detracts from appreciating the implications from the findings.

A:Dear Professor, thank you for your advice. We have added the study design and how location locations-sites/plots/samples/time intervals in the Materials and monitor system section.

Q3:Please add to Table 1 a blank row to provide clarity and space - between the "Primary division" categories and for Table 4 adding a blank row to provide clarity and space - between the "Secondary division" categories.

A:Dear Professor, thank you for your advice. We have added enough space in every row in these table to improve the clarity and space between each categories.

Q4:The information of Figure 2 and Figure 3

A:Dear Professor, this advice is not a completed one. But we also checked these figure in our draft to avoid some obvious mistakes.
